# Short-term outcomes after minimally invasive versus open pancreaticoduodenectomy in elderly patients: a propensity score-matched analysis

**DOI:** 10.1186/s12893-021-01052-2

**Published:** 2021-01-25

**Authors:** Shih-Min Yin, Yueh-Wei Liu, Yu-Yin Liu, Chee-Chien Yong, Chih-Chi Wang, Wei-Feng Li, Cheng-Hsi Yeh

**Affiliations:** Division of General Surgery, Department of Surgery, Kaohsiung Chang Gung Memorial Hospital and Chang Gung University College of Medicine, No.123, DAPI Rd. Niaosng Dist, Kaohsiung City, 83301 Taiwan

**Keywords:** Minimally invasive pancreatoduodenectomy, Elderly patients, Short-term postoperative outcomes, Propensity score-matched analysis

## Abstract

**Background:**

To date, the evidence on the safety and benefits of minimally invasive pancreatoduodenectomy (MIPD) in elderly patients is still controversy. This study aim to compare the risk and benefit between MIPD and open pancreatoduodenectomy (OPD) in elderly patients.

**Methods:**

From 2016 to 2020, we retrospective enrolled 26 patients underwent MIPD and other 119 patients underwent OPD. We firstly compared the baseline characteristics, 90-day mortality and short-term surgical outcomes of MIPD and OPD. Propensity score matching was applied for old age patient (≥ 65-year-old vs. < 65-year-old) for detail safety and feasibility analysis.

**Results:**

Patients received MIPD is significantly older, had poor performance status, less lymph node harvest, longer operation time, less postoperative hospital stay (POHS) and earlier drain removal. After 1:2 propensity score matching analysis, elderly patients in MIPD group had significantly poor performance status (P = 0.042) compared to OPD group. Patients receiving MIPD had significantly shorter POHS (18 vs. 25 days, P = 0.028), earlier drain removal (16 vs. 21 days, P = 0.012) and smaller delay gastric empty rate (5.9 vs. 32.4% P = 0.036). There was no 90-day mortality (0% vs. 11.8%, P = 0.186) and pulmonary complications (0% vs. 17.6%, P = 0.075) in MIPD group, and the major complication rate is comparable to OPD group (17.6% vs. 29.4%, P = 0.290).

**Conclusion:**

For elderly patients, MIPD is a feasible and safe option even in patients with inferior preoperative performance status. MIPD might also provide potential advantage for elderly patients in minimizing pulmonary complication and overall mortality over OPD.

## Background

According to the latest Global Health Observatory data of the World Health Organization (2016), the average life expectancy of the global population is 72 years. Despite reduction in mortality rates due to improved surgical techniques, elderly patients are still considered a high-risk population for major abdominal surgery [1, 2]. On the other hand, since the increase in life expectancy may be associated with the risk of developing periampullary cancer [3–5], it is reasonable to expect higher number of elderly patients with resectable periampullary cancer.

Pancreaticoduodenectomy (PD) is considered the only potentially curative surgical procedure in patients with periampullary malignancy. However, it is also one of the most challenging and complex surgeries due to requirement of numerous reconstructions and the presence of anatomical variations. Although mortality rates for pancreatic surgery in high volume centers can be much lower (approximately 3%) [6], several studies still reported relatively high mortality (up to 6%) and morbidity (ranging approximately 40–50% [7, 8], including common complications such as pancreatic fistulas, post-pancreatectomy bleeding, anastomotic leakage and delayed gastric emptying. Therefore, performing PD in elderly patients is challenging and controversial. Although several studies have reported on the safety and feasibility of open pancreaticoduodenectomy (OPD) in elderly patients without significant increase in the mortality and morbidity [9, 10], the risk for major complication and prolong postoperative recovery is still remain great concern. Recent studies have also reported more number of major post-pancreatectomy complications [11], and significantly higher 30-day and 60-day mortality in elderly patients [12, 13].

Minimally invasive surgery (MIS) has been accepted for the treatment of gastrointestinal and colorectal malignancies owing to its safety and feasibility, even in elderly patients [14, 15]. However, minimally invasive pancreaticoduodenectomy (MIPD), including laparoscopic pancreaticoduodenectomy (LPD) and robotic pancreaticoduodenectomy (RPD), are not routinely performed due to technique sensitivity and safety considerations involved. Although several meta-analysis and case series have reported the safety and feasibility of MIPD compared to OPD [8, 16–18], evidence from randomized controlled trials remain controversial [19, 20]. In particular, there is limited evidence regarding safety and benefits of MIPD compared to OPD in elderly patients.

The aim of this study was to compare the short-term postoperative outcomes of MIPD and OPD between elderly (≥ 65-years-old) and non-elderly (< 65-years-old) patients. By using propensity score-matching (PSM) analysis, we aimed to precisely evaluate the safety and feasibility of these procedures in elderly patients.

## Methods

### Patient selection and choice of surgical procedure

**Fig. 1:**
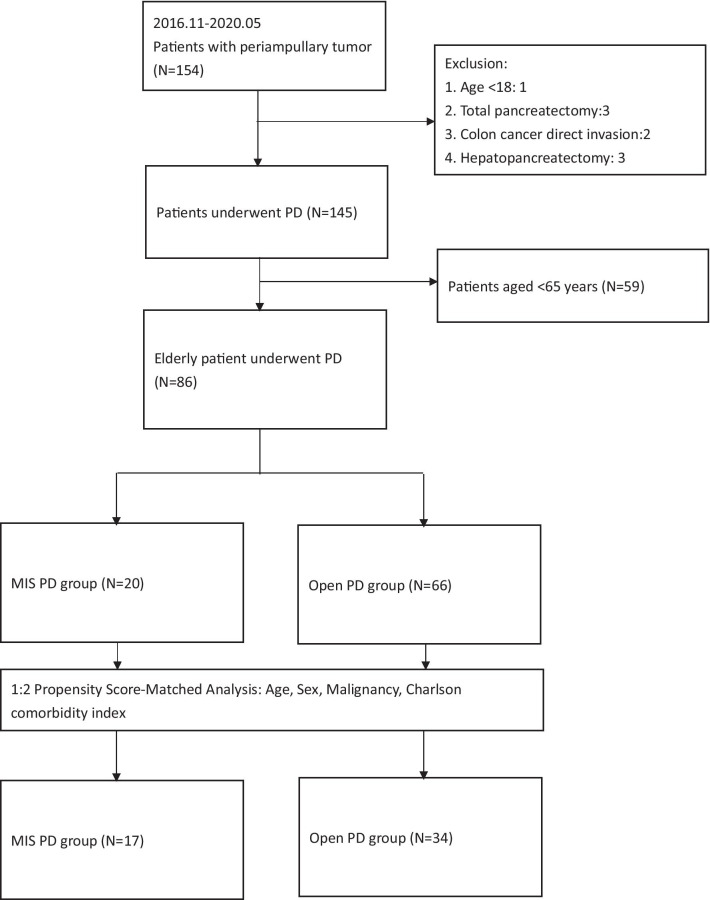
Flow chart of the patient selection process. *PD* pancreaticoduodenectomy, *OPD* open pancreaticoduodenectomy, *MIPD* minimal invasive pancreaticoduodenectomy

In the present study, we retrospectively collected clinical data of patients who underwent PD for periampullary tumors between November 2016 and May 2020. The study was approved by the Institutional Review Board (IRB), under authorization No. 202000247B0, and was conducted at Kaohsiung Chang Gang Memorial Hospital, Kaohsiung, Taiwan. The patients were divided into two groups based on the surgical approach used. Age cut-off of 65 years was considered in the definition of “Elderly patients,” which was compatible with previous studies on pancreatic surgery in elderly patients [21, 22]. Patient less than 18-year-old, PD was performed due to colon cancer direct invasion, and patients converted to total pancreatectomy or hepatopancreatectomy were excluded. The study flow diagram was shown in Fig. 1

The patients made the choice of minimal invasive surgery (including LPD and RPD) or traditional open approach after their attending doctors carefully explained the surgical details, advantages and disadvantages, and the possible cost difference between the different approaches. We did not consider patients with previous laparotomic surgery as the contraindication for MIPD candidates. However, for patient underwent neoadjuvant chemotherapy and patients with borderline resectable pancreatic cancer, the traditional open approach would be suggest to the patients. The same group of surgeons performed the surgery, and patients received the standard postoperative care and surveillance protocol.

### Surgical procedures

Both LPD and RPD were performed by the hybrid method. The laparoscopic procedure was started with three 12 mm ports and two 5 mm ports. The robotic procedure was started with three robotic 8 mm ports, one 12 mm camera port, and one 12 mm assistant port. The dissection was done with the “Artery first” approach from left to right, bottom to top. Standard pancreatoduodenectomy (classical Whipple procedure) was performed. In cases with malignancy, extended lymph node dissection and level II total mesopancreas excision was done. After complete resection of the specimen, hepatojejunostomy and retrocolic gastrojejunostomy were performed by the minimally invasive method. A 5–7 cm upper midline incision was made to remove the specimen and perform pancreaticojejunostomy by hand-sewing, duct to mucosa method. If severe adhesion or tumor invasion of a major vessel was encountered, vascular resection and reconstruction was also performed by the hybrid method from the upper midline wound at the end of resection. All OPD surgeries were performed by the classical Whipple procedure.

### Surgical and postoperative outcomes

The overall morbidity and in-hospital mortality were considered the primary outcome measures. Preoperative data of the Eastern Cooperative Oncology Group (ECOG) Scale of Performance Status, American Society of Anesthesiologists (ASA) physical status classification, and other comorbidities were compared between MIPD and OPD groups. Charlson comorbidity index (CCI) was also applied for comorbidity evaluation [23]. Other surgical data including operative time, intra-operative estimated blood loss (EBL), and conversion rate was recorded. Postoperative recovery data including postoperative hospital stay (POHS), intensive care unit (ICU) stay, time of initiation of diet, ambulation, and complete drain removal was also compared between the groups. Recorded post-operative complications included postoperative pancreatic fistula (POPF), delayed gastric emptying (DGE), post-pancreatectomy hemorrhage (PPH), pulmonary complication and intraabdominal infection (IAI) among other major complications. POPF, DGE, and PPH were defined and classified by the International Study Group of Pancreatic Surgery (ISGPS) [24–26], and only grade B and grade C POPF were recorded. A major complication was considered as one with a score ≥ 3 based on Clavien–Dindo classification [27]. Mortality was defined as death before discharge of the patient or within 90 days after surgery.

### Statistical analyses

Statistical analyses were performed using IBM SPSS Statistics for Windows, version 20.0 (IBM Corp., Armonk, NY, USA) and NCSS 10 software (NCSS Statistical Software, Kaysville, UT, USA). Two-sided Fisher’s exact or Pearson chi-square tests were used to compare categorical data. The normally distributed continuous and non-normally distributed data were analyzed with unpaired Student’s t- and Mann–Whitney U-tests, respectively, and presented as mean ± standard deviation or median with interquartile rage (IQR).

To minimize the potential confounding effects of the compared patient populations due to non-randomized assignment, a 1:2 PSM study group (MIS vs. Open surgery) was created using the Greedy method with a 0.2 caliper width using NCSS 10 software. The PSM analysis was performed using a logistic regression model with the following covariates: Age, sex, malignancy status, and CCI. After adjusting for these confounding factors, binary logistic regression analysis was used to evaluate the effect of minimally invasive and open surgery on postoperative recovery. Statistical significance was set at a P-value of < 0.05 for each analysis.

## Results

### Baseline characteristics and clinicopathological variables of patients

We totally enrolled 154 patients with periampullary tumor in this retrospective series. After excluded 1 patient less than 18-year-old, 2 patients with colon cancer direct invasion, 3 patients converted to total pancreatectomy and 3 patient received hepatopancreatectomy, total 145 patients were eligible for our further analysis (Fig. 1). Out of the total 145 patients included in this retrospective series, 119 patients were in the OPD group, and 26 patients were in the MIPD group (six patients underwent RPD and 20 patients underwent LPD). Baseline characteristics and clinicopathological outcomes are presented in Table 1. The mean age of patients in MIPD group was higher than those in the OPD group (71.03 ± 8.8 and 64.40 ± 11.7 years, P = 0.008). Patients in the MIPD group also had poor performance status (P = 0.036) and less number of harvested lymph nodes (14.23 ± 8.04 vs. 19.83 ± 9.71, P = 0.04). Similar male-to-female ratio, ASA, tumor size, previous abdominal surgery, rate of preoperative biliary drainage, and underlying comorbidity was found between two groups. Details of pathologic outcomes are also listed in Table 1.

**Table 1:** Overall baseline characteristics and pathological outcomes

Variables	MIPD (N = 26)	OPD (N = 119)	P-value
Age (years)	71.03 ± 8.8	64.40 ± 11.70	*0.008*
Male (Male)	16 (61.5%)	70 (58.8%)	0.490
ASA (n, %)			0.895
I	0 (0%)	1 (0.8%)	
II	10 (38.5%)	46 (38.7%)	
III	16 (61.5%)	72 (60.5%)	
ECOG (n, %)			*0.003*
0	19 (73.1%)	112 (94.1%)	
1	5 (19.2%)	6 (5.0%)	
2	2 (7.7%)	1 (0.8%)	
Pathology (n, %)			*0.036*
Benign	1 (3.8%)	9 (7.6%)	
Ampullary cancer	13 (50.0%)	25 (21.0%)	
CBD cancer	4 (15.4%)	17 (19.3%)	
PDAC	5 (19.2%)	40 (42.0%)	
IPMN	2 (7.7%)	3 (2.5%)	
PNET	0 (0%)	6 (5.0%)	
Duodenal cancer	1 (3.8%)	3 (2.5%)	
Tumor size (cm ± SD)	2.68 ± 1.80	3.14 ± 1.33	0.231
Lymph node harvest (number ± SD)	14.23 ± 8.04	19.83 ± 9.71	*0.004*
Pre-op bile drain (n, %)	14 (53.8%)	70 (58.8%)	0.400
Abdominal surgery history (n, %)	5 (19.2%)	25 (21.0%)	0.539
Hypertension (n, %)	14 (53.8%)	50 (42.0%)	0.189
DM (n, %)	8 (30.8%)	26 (21.8%)	0.232
CAD (n, %)	1 (3.8%)	15 (12.6)	0.174
COPD (n, %)	1 (3.8%)	2 (1.7%)	0.450
CKD (n, %)	1 (3.8%)	10 (8.4%)	0.378
CCI (n, %)			0.062
0	8 (30.8%)	63 (52.9%)	
1	10 (38.5%)	36 (30.3%)	
2	7 (26.9)	16 (13.4%)	
3	0 (0%)	3 (2.5%)	
4	0 (0%)	1 (0.8)	
5	1	0 (0%)	

Italic indicate statistic significance

*MIPD* minimal invasive pancreatoduodenectomy, *OPD* open pancreatoduodenectomy, *ASA score* American Society of Anesthesiologists classification score, *ECOG* Eastern Cooperative Oncology Group, *CBD* Common bile duct, *PDAC* pancreatic ductal adenocarcinoma, *IPMN* intraductal papillary mucinous neoplasm, *PNET* pancreatic neuroendocrine tumor, *DM* Diabetes Mellitus, *CKD* chronic kidney disease, *CCI* Charlson comorbidity index

### Short-term postoperative outcomes of patients

Table 2 shows surgical data and short-term postoperative outcomes of the two groups. Patients in the MIPD group had significantly longer surgical time (540 vs. 462 min, P = 0.011), shorter POHS (18 vs. 24 days, P = 0.001), and earlier complete drain removal (16 days vs. 22 days, P < 0.001). The overall conversion rate in the MIPD group was 11.1%. There was no significant difference in EBL, postoperative ICU stay, time of initiation of oral diet and ambulation, total parenteral nutrition (TPN) dependence, and 30-days re-admission rate. Occurrence of surgical mortality, major complications and rates of POPF, DGE, and PPF were similar between OPD and MIPD groups.

**Table 2:** Overall short-term surgical outcomes

Variables	MIPD (N = 26)	OPD (N = 119)	P-value
EBL (mL, IQR)	300 (87–562)	300 (150–400)	0.981
Operation time (min, IQR)	540 (420–662)	462 (370–596)	*0.011*
Conversion (n, %)	3 (11.5%)	–	–
P duct size (mm ± SD)	3.27 ± 1.41	3.57 ± 2.04	0.958
POHS (days, IQR)	18 (14–26)	24 (19–33)	*0.001*
Initiate oral diet (days, IQR)	5 (3–6)	5 (3–6)	0.676
Ambulation (days, IQR)	6 (4–7)	6 (4–7)	0.815
Drain removal (days, IQR)	16 (12–20)	22 (16–30)	< *0.001*
ICU stay (days, IQR)	3 (2–6)	3 (2–6)	0.743
TPN dependence (days, IQR)	7 (5–11)	8 (6–12)	0.184
Major complication (≥ CD Gr. 3)	3 (11.5%)	27 (22.7%)	0.157
PPH (Grade B and C)	2 (7.7%)	10 (8.4%)	0.633
DGE (Grade B and C)	2 (7.7%)	28 (24.6%)	0.071
POPF (Grade B and C)	2 (7.7%)	26 (21.8%)	0.077
Pulmonary complication	0 (0%)	10 (8.4%)	0.129
IAI	5 (19.2%)	40 (33.6%)	0.113
30-day readmission	1 (3.8%)	7 (5.8%)	0.865
Mortality	0 (0%)	6 (5.0%)	0.299

Italic indicate statistic significance

*EBL* estimated blood loss, *IQR* interquartile range, SD standard deviation, *POHS* post-operative hospital stay, *ICU* intensive care unit, *TPN* total parenteral nutrition, CD Gr. Clavien–Dindo grade, *PPH* post-pancreatectomy hemorrhage, *DGE* delayed gastric emptying, *POPF* post-operative pancreatic fistula, *IAI* intraabdominal infection

### Overall comparison between MIPD and OPD in elderly patients

The baseline characteristics and short-term postoperative outcomes in elderly patients are presented in Tables 3 and 4. In this cohort, 20 elderly patients underwent MIPD while 66 underwent OPD. Significantly shorter POHS (18 days vs. 24 days, P = 0.014) and earlier complete drain removal (16 days vs. 22 days, P = 0.004) was observed in elderly patients who underwent MIPD. The occurrence of DGE was significantly less in MIPD group (10% vs. 33%, P = 0.042). There was no significant difference in parameters of sex, ASA score, EBL, harvested lymph nodes, and other short-term postoperative outcomes. Similar rate of major complications, POPF and PPF was found between two groups. No death was reported in elderly patients who underwent MIPD; however, the mortality rate in the OPD group was 9.1% (P = 0.193).

**Table 3:** Baseline characteristics and pathological outcomes in elderly patients: PSM analysis

Variables	Original cohort		P-value	Matched cohort		P-value
	MIPD (N = 20)	OPD (N = 66)		MIPD (N = 17)	OPD(N = 34)	
Age (years)	74.5 ± 6.77	72.48 ± 5.35	0.234	73.76 ± 5.68	73.70 ± 6.82	0.974
Male (Male)	14 (70.0%)	40 (60.6%)	0.313	13 (76.5%)	25 (73.5%)	0.553
Malignancy (n, %)	19 (95%)	59 (84.9%)	0.402	16 (94.1%)	32 (94.1%)	> 0.999
CCI (n, %)			0.211			0.558
0	6 (30.0%)	28 (42.4%)		6 (35.3%)	12 (35.3%)	
1	6 (30.0%)	22 (33.3%)		6 (35.3%)	12 (35.3%)	
2	7 (35.0%)	12 (18.2%)		4 (23.5%)	8 (23.5%)	
3	0 (0%)	3 (4.5%)		0 (0%)	2 (5.9%)	
4	0 (0%)	1 (1.5%)		0 (0%)	0 (0%)	
5	1 (5.0%)	0 (0%)		1 (5.9%)	0 (0%)	
ASA (n, %)			0.388			0.463
I	0 (0%)	0 (0%)		0 (0%)	0 (0%)	
II	5 (25.0%)	21 (31.8%)		4 (23.5%)	10 (29.4%)	
III	15 (75.0%)	45 (68.2%)		13 (76.5%)	24 (70.6%)	
Tumor type (n, %)			0.402			0.583
Benign	1 (5.0%)	6 (9.1%)		1 (3.8%)	2 (5.9%)	
Ampullary cancer	9 (45.0%)	16 (24.2%)		7 (41.2%)	10 (29.4%)	
CBD cancer	4 (20.0%)	11 (16.7%)		3 (17.6%)	8 (23.5%)	
PDAC	5 (25.0%)	26 (39.4%)		5 (29.4%)	12 (35.3%)	
IPMN	0 (0%)	3 (4.5%)		0 (0%)	2 (5.9%)	
PNET	0 (0%)	3 (4.5%)		0 (0%)	0 (0%)	
Duodenal cancer	1 (5.0%)	1 (1.5%)		1 (5.9%)	0 (0%)	
ECOG (n, %)			*0.008*			*0.042*
0	13 (65.0%)	61 (92.4%)		12 (70.6%)	32 (94.1%)	
1	5 (25.0%)	4 (6.1%)		3 (17.6%)	2 (5.9%)	
2	2 (10%)	1 (1.5%)		2 (11.8%)	0 (0%)	
Tumor size (cm ± SD)	2.98 ± 1.93	3.26 ± 1.49	0.562	2.8. ± 2.0	2.92 ± 1.25	0.844
Lymph node harvest (number ± SD)	15.35 ± 8.26	19.42 ± 9.88	0.075	16.94 ± 8.56	17.44 ± 7.77	0.840
Pre-op bile drain (n, %)	8 (66.7%)	39 (59.1%)	0.696	10 (58.8%)	21(61.8%)	0.537
Abdominal surgery history (n, %)	4 (20.0%)	13 (19.7%)	0.601	3 (17.6%)	8 (23.5%)	0.462

Italic indicate statistic significance

*PSM* propensity score matching, *MIPD* minimal invasive pancreatoduodenectomy, *OPD* open pancreatoduodenectomy, *CCI* Charlson comorbidity index, *ASA score* American Society of Anesthesiologists classification score, *ECOG* Eastern Cooperative Oncology Group

**Table 4:** Short-term surgical outcomes in elderly patients: PSM analysis

Variables	Original cohort		P-value	Matched cohort		P-value
	MIPD (N = 20)	OPD (N = 66)		MIPD (N = 17)	OPD (N = 34)	
EBL (mL, IQR)	275 (100–687)	300 (150–462)	0.822	200 (50–500)	300 (150–462)	0.880
Operation time (min, IQR)	525 (414–640)	467 (377–604)	0.161	420 (410–526)	456 (360–604)	0.181
P duct size (mm ± SD)	3.2 ± 1.46	3.6 ± 1.87	0.51	2.88 ± 1.30	3.66 ± 1.69	0.104
POHS (days, IQR)	18 (14–27)	24 (19–33)	*0.014*	18 (14–28)	25 (18–33)	*0.028*
Initiate oral diet (days, IQR)	5 (3–7)	5 (4–6)	0.946	5 (3–7)	5 (4–6)	0.992
Ambulation (days, IQR)	7 (5–8)	6 (5–9)	0.922	7 (5–8)	6 (5–10)	0.739
Drain removal (days, IQR)	16 (13–20)	22 (17–27)	*0.004*	16 (13–20)	21 (17–27)	*0.012*
ICU stay (days, IQR)	4 (3–6)	4 (3–7)	0.909	4 (3–6)	4 (3–7)	0.840
TPN dependence (days, IQR)	8 (3–11)	9 (6–14)	0.264	8 (4–11)	10 (7–15)	0.167
Major complication (≥ CD Gr. 3)	3 (15.0%)	20 (30.3%)	0.142	3 (17.6%)	10 (29.4%)	0.290
PPH (Grade B and C)	2 (10.0%)	8 (12.1%)	0.577	2 (11.8%)	3 (8.8%)	0.546
DGE (Grade B and C)	2 (10.0%)	22 (33.3%)	*0.042*	1 (5.9%)	11 (32.4%)	*0.036*
POPF (Grade B and C)	2 (10.0%)	14 (21.2%)	0.217	2 (11.8%)	8 (23.5%)	0.273
Pulmonary complication	0 (0%)	10 (15.2%)	0.06	0 (0%)	6 (17.6%)	0.075
IAI	5 (25.0%)	19 (28.8%)	0.491	5 (29.4%)	11 (32.4%)	0.491
30-day readmission	1 (5.0%)	3 (4.5%)	0.856	1 (5.9%)	2 (5.9%)	> 0.999
Mortality	0 (0%)	6 (9.1%)	0.193	0 (0%)	4 (11.8%)	0.186

Italic indicate statistic significance

*EBL* estimated blood loss, *IQR* interquartile range, SD standard deviation, *POHS* post-operative hospital stay, *ICU* intensive care unit, *TPN* total parenteral nutrition, CD Gr. Clavien–Dindo grade, *PPH* post-pancreatectomy hemorrhage, *DGE* delayed gastric emptying, *POPF* post-operative pancreatic fistula, *IAI* intraabdominal infection

### Propensity score-matched comparison of elderly patients who underwent MIPD and OPD

A one-to-two PSM analysis was applied (17 patients in the MIPD group and 34 patients in the OPD group), adjusted for sex, age, CCI and pathologies, as shown in Table 3. Although poor preoperative performance status was observed in patients underwent MIPD (P = 0.042), the MIPD group still presented significantly shorter POHS (18 days vs. 25 days, P = 0.028), earlier complete drain removal (16 days vs. 21 days, P = 0.012) and less rate of DGE (5.9% vs. 34.2%, P = 0.036) than the OPD group. There were no significant differences in surgical time, EBL, initiation of oral diet and ambulation, postoperative ICU stay, and re-admission rate. Despite the analysis did not achieve statistical significance, the pulmonary complications (0% in the MIPD group vs. 17.6% in the OPD group, P = 0.075) and mortality (0% in the MIPD group vs. 11.8% in the OPD group, P = 0.186) tended to be more common in the OPD group.

### Analysis of six surgical mortality cases in OPD group

The details of six mortality cases in our study were revealed in Table 5. All of our mortality cases were over 65-year-old. 3 patients was died of pulmonary complication (2 Nosocominal pneumonia and 1 aspiration pneumonia), 2 patients died of IAI with septic shock and 1 died of uncontrolled PPH. Relatively longer mean operation time (507.5 min), increased mean EBL (591.6 mL), high DGE rate, high POPF rate and late ambulation was also observed in these cases.

**Table 5:** Analysis of six surgical mortality cases in OPD group

Case	Age	Sex	Disease	CCI	ECOG	OPT (mins)	EBL (mL)	DGE	POPF (Grade)	Amulation (day)	Cause of death
1	80–90	1	PDAC	2	0	656	450	None	0	None	Pneumonia
2	80–90	1	pNET	2	0	562	400	Yes	0	10	Pneumonia
3	80–90	1	CBD cancer	1	0	340	250	None	3	6	PPH
4	70–80	1	PDAC	0	1	607	1000	None	0	4	IAI with septic shock
5	70–80	2	Duodenum cancer	2	2	270	350	Yes	3	None	IAI with septic shock
6	80–90	1	Pancreatitis	1	0	610	1100	Yes	0	10	Pneumonia

*OPD* open pancreatoduodenectomy, *PDAC* pancreatic ductal adenocarcinoma, *PNET* pancreatic neuroendocrine tumor, *CBD* common bile duct, *OPT* operation time, *CCI* Charlson comorbidity index, *EBL* estimated blood loss, *ECOG* Eastern Cooperative Oncology Group, *DGE* delayed gastric emptying, *POPF* post-operative pancreatic fistula, *PPH* post-pancreatectomy hemorrhage, *IAI* intraabdominal infection

## Discussion

In our retrospective study, we compared the postoperative outcomes between MIPD and OPD in elderly patients over 65 years of age. We also conducted a 1:2 PSM analysis to precisely evaluate the safety and efficacy of MIPD in elderly patients and minimize the non-randomization bias in the results. Elderly patients who underwent MIPD had significantly shorter POHS, earlier drain removal and less DGE than those in the OPD group, the results of which were consistent before and after the PSM analysis. The time of initiation of oral diet, ICU stay, TPN dependence, and time taken for ambulation were comparable between the groups. Many recent studies have reported that MIPD enabled early recovery, reduced postoperative pain and the need for analgesic injections and shortened the duration of POHS [28–30]. We also found MIPD is not commonly associated with delay gastric empty, which is a significant complication after OPD in elderly patients. Although the reason for the decreasing rate of delayed gastric emptying by MIPD is still under investigation, current studies had support that minimal invasive surgery with less tissue trauma might play a role [30, 31]. Despite of the reported benefit, MIPD did not completely mitigate the postoperative risk in elderly patients, and evidence regarding the benefits of the approach in this population remains limited. Our study confirmed the potential benefit of MIPD in improving recovery in elderly patients, without increasing the rate of perioperative complications and mortality.

PD is one of the most challenging surgeries owing to technique sensitivity, proximity of major vascular structures, and occurrence of various postoperative complications. Previous studies have reported that the 90-day mortality following PD in elderly patients may exceed 10%, with the overall rate of morbidity ranging from 40 to 50% regardless of OPD or MIPD [32, 33]. In our series, there was no mortality in the MIPD group, and the rate of major complications was 15.0%, which was notably lower than in the OPD group (30.3%, P = 0.142) for elderly patients. We also observed that the MIPD group had fewer rates of pulmonary complications and post-pancreatectomy bleeding. The P value between the two groups was not significantly different, which might be due to the small sample size of the study. Among the six deaths in the OPD group, only one was due to POPF related hemorrhage. Other three deaths were due to postoperative pneumonia secondary to late ambulation or aspiration. Significantly less DEG rate in MIPD group might explain the lower risk of aspiration for elderly patients, which leading less pulmonary complication in our series. Although our data was inadequate to provide strong evidence regarding prevention of pulmonary complications by MIPD, several studies have reported that reducing postoperative pain by MIS may lead to decreased incidence of pneumonia [34, 35], which may explain the absence of mortality in the MIPD group.

Several studies have demonstrated that the risk of postoperative mortality and complications following MIPD may be higher in low volume centers (≤ 22–25 cases per year) [36, 37]. In our study, all MIPD was performed by at least two experienced hepato-pancreatico-biliary attending surgeon. We also decided to perform the surgery by the hybrid method to minimize the risk of POPF, which is the most common complication of PD. In instances of significant bleeding or requirement of vascular reconstruction during MIPD, early open conversion (11.1%) was done. Notably, elderly patients in the MIPD group had significantly poor preoperative grade on ECOG scale; however, the surgical mortality and incidence of major complications were similar and even lower than in the OPD group. Our study proposed that MIPD is safe and feasible for elderly patients in low to moderate volume centers like our hospital through proper selection of candidates and by utilizing the hybrid method. The results of our study were comparable to those reported in the literature.

Several potential limitations of this study should be considered. Although we applied the PSM analysis to minimize the bias of the retrospective and non-randomized data, our study only considered short-term postoperative outcomes in a small cohort of elderly patients. Further large sample, prospective, and randomized controlled studies should be performed including long-term survival in order to confirm the results of the current study. Another limitation of the study is that our center started LPD in 2015, performing around 10–12 cases annually. Studies have reported that hepato-pancreatico-biliary surgeons might be required to perform more than 30 cases of LPD [38, 39] and 20 cases of RPD [40] to overcome the learning curve; therefore, the advantages of MIPD in elderly patients might be underestimated from our current data.

## Conclusion

In conclusion, our study demonstrated that MIPD, compared to OPD, is a feasible and acceptable alternative for elderly patients with periampullary tumors. After counterbalancing the difference for patients and tumor-related characteristics, MIPD still have some advantages over OPD such as shorter hospital stay, earlier drain removal and less DGE rate. The trend towards reduced pulmonary complication and mortality observed in our study might be confirmed in more precise randomized controlled studies.

## Data Availability

All data generated by and used in the study is available from the corresponding author upon reasonable request.

## Abbreviations

PD: Pancreaticoduodenectomy
OPD: Open pancreaticoduodenectomy
MIS: Minimally invasive surgery
MIPD: Minimally invasive pancreaticoduodenectomy
LPD: Laparoscopic pancreaticoduodenectomy
RPD: Robotic pancreaticoduodenectomy
PSM: Propensity score-matching (PSM)
ECOG: Eastern Cooperative Oncology Group
ASA: American Society of Anesthesiologists
CCI: Charlson comorbidity index
EBL: Estimated blood loss
POHS: Postoperative hospital stay
ICU: Intensive care unit
POPF: Postoperative pancreatic fistula
DGE: Delayed gastric emptying
PPH: Post-pancreatectomy hemorrhage
IQR: Interquartile rage
IAI: Intraabdominal infection
